# Effects of therapeutic zinc supplementation for diarrhea and two preventive zinc supplementation regimens on the incidence and duration of diarrhea and acute respiratory tract infections in rural Laotian children: A randomized controlled trial

**DOI:** 10.7189/jogh.10.010424

**Published:** 2020-06

**Authors:** Maxwell A Barffour, Guy-Marino Hinnouho, K Ryan Wessells, Sengchanh Kounnavong, Kethmany Ratsavong, Dalaphone Sitthideth, Bangone Bounheuang, Khanpaseuth Sengnam, Bigphone Chanhthavong, Charles D Arnold, Kenneth H Brown, Charles P Larson, Sonja Y Hess

**Affiliations:** 1 Institute for Global Nutrition, University of California, Davis, California, USA; 2 College of Health and Human Services, Public Health Program, Missouri State University, Springfield, Missouri, USA; 3 Lao Tropical and Public Health Institute, Vientiane, Lao People’s Democratic Republic; 4 School of Population and Global Health, McGill University, Montreal, Canada

## Abstract

**Background:**

Diarrhea and respiratory tract infections are leading causes of childhood morbidity and mortality. This individually randomized, double-blind placebo-controlled trial was designed to evaluate the effects of different zinc supplementation regimens on the incidence and duration of diarrhea and acute lower (ALRI) and upper (AURI) respiratory tract infections among rural Laotian children. The study included 3407 children, 6-23 months at enrollment.

**Methods:**

Children were randomized to one of four study groups: therapeutic zinc supplements for diarrhea treatment (20 mg/d for 10 days with each episode; TZ), daily preventive zinc tablets (7 mg/d; PZ), daily multiple micronutrient powder (10 mg/d zinc, 6 mg/d iron and 13 other micronutrients; MNP), or daily placebo powder for 9 months. Incidence and duration of diarrhea (≥3 liquid stools/24 hours), ALRI (persistent cough with wheezing, stridor or chest in-drawing) and AURI (purulent nasal discharge with cough) were assessed by parental report during weekly home visits and analyzed using negative binomial models.

**Results:**

Baseline mean age was 14.2 ± 5.1 months, and 71% had low plasma zinc (<65 μg/dL). Overall diarrhea incidence (0.61 ± 0.01 episodes/100 days at risk) and duration (2.12 ± 0.03 days/episode) did not differ by study group. Age modified the impact of the interventions on diarrhea incidence (*P* = 0.06) and duration (*P* = 0.01). In children >18 months, TZ reduced diarrhea incidence by 24% vs MNP (*P* = 0.035), and 36% vs Control (*P* = 0.004), but there was no difference with PZ. This patterned remained when analyses were restricted to diarrhea episode occurring after the first treatment with TZ. Also, in children >18 months, TZ reduced diarrhea duration by 15% vs PZ (*P* = 0.03), and 16% vs Control (*P* = 0.03), but there was no difference with MNP. There were no overall effects of study group on incidence of ALRI (overall mean 0.005 ± 0.001 episodes/100 days, *P* = 0.14) or AURI (overall mean 0.09 ± 0.01 episodes/100 days, *P* = 0.72).

**Conclusions:**

There was no overall impact of TZ, PZ or MNP on diarrhea, ALRI and AURI. However, in children >18 months, TZ significantly reduced both the duration of diarrhea episodes and the incidence of future diarrhea episodes compared with placebo.

**Trial registration:**

ClinicalTrials.gov: NCT02428647.

Each year, ~ 700 000 children less than five years of age worldwide die from diarrhea, and ~ 1.3 million die from pneumonia. A disproportionately high percentage of these deaths (>90%) occurs in low and middle income countries [1], where zinc deficiency is prevalent [2-4]. In children 6 months and older, adjunctive treatment of diarrhea with zinc has been shown to reduce the duration of illness and decrease the likelihood of progression to persistent diarrhea [5]. Limited evidence also suggests that therapeutic zinc supplementation for diarrhea may prevent new episodes of diarrhea in the 2-3 months following initial treatment [6]. Thus, the World Health Organization (WHO) and UNICEF recommend therapeutic zinc (20 mg), to be given daily for 10-14 days during diarrhea episodes, along with oral rehydration salt (ORS) solution (given on diarrhea days) [7,8]. Unfortunately, restricting zinc supplementation just for diarrhea treatment has several potential limitations, because this strategy requires appropriate recognition of diarrhea, caregiver motivation to seek treatment and access to a health care facility or pharmacy to obtain the supplements. Coverage of therapeutic zinc supplementation programs is often low, typically reaching <30% of children in need [9,10]. In addition, this strategy does not target other micronutrient deficiencies and has not been shown to affect other functional outcomes related to zinc deficiency, such as physical growth and risk of pneumonia [11-14]. Thus, a preventive zinc supplementation approach, universally targeting all children at risk for zinc deficiency, may be a better strategy.

Meta-analyses of preventive zinc-alone supplementation trials found a 20% reduction in the incidence of diarrhea and a 14% reduction in the incidence of respiratory tract infection; with the beneficial effects restricted to children >12 months of age [2], although there was significant heterogeneity across trials. Additional evidence is needed to compare the impact of preventive vs therapeutic zinc supplementation in the same population.

Uncertainties remain regarding the optimal formulation of preventive zinc supplements. When delivered as a single micronutrient supplement, zinc has been shown to improve zinc status, increase linear growth and weight gain, and reduce the burden of diarrhea and respiratory infections [2,15-17]. However, because zinc deficiency often coexists with several other micronutrient deficiencies, multiple micronutrient formulations, such as multiple micronutrient powders (MNP), are generally preferred for programmatic interventions. The WHO and UNICEF currently promote an MNP formulation containing 12.5 mg iron, 4.1-5 mg zinc and 13 other micronutrients [18]. Despite the proven efficacy of MNP to prevent iron deficiency and anemia [19], its effect on morbidity is uncertain, primarily because there is little evidence from randomized controlled trials and the available results are inconsistent. In Pakistani children, MNP was associated with an increased incidence of acute and bloody diarrhea, but there was no overall effect on hospital admission due to diarrhea, respiratory illness or febrile episodes [20]; and a study in Ghana found a higher rate of hospitalization among children who received MNP [21]. It has been speculated that these reported adverse effects may be related to the iron contained in the MNP formulation [22]. In light of these observations, in the present study, we used a new MNP formulation, which contained a lower amount of iron (6 mg per daily dose) and a higher amount of zinc (10 mg per daily dose) than current formulations, along with 13 other micronutrients.

The present study was designed to assess the effects of therapeutic zinc supplementation (20 mg zinc/d for 10 days, TZ) for the treatment of diarrhea, daily preventive zinc supplementation delivered alone (7 mg zinc/d, PZ), and a daily preventive MNP (containing 10 mg zinc, 6 mg iron and 13 other micronutrients, MNP) on the incidence and duration of diarrhea in rural Laotian children. As secondary outcomes, we also assessed treatment effects on acute lower or upper respiratory tract illnesses.

## METHODS

### Ethical approval

The protocol of this trial, known as the Lao Zinc Study, and the consent procedures were approved by the National Ethics Committee for Health Research (NECHR), Ministry of Health, Lao People’s Democratic Republic (PDR), and the Institutional Review Board of the University of California, Davis, USA. The trial is registered as a clinical trial with the US National Institutes of Health (ClinicalTrials.gov; NCT02428647). The full study protocol has been published previously [23].

### Study design and participants

This double-blind, randomized, placebo-controlled trial was implemented from September 2015 through April 2017 in rural communities in Khammouane Province, Lao PDR. This province was chosen because of its high prevalence of stunting [24], which is suggestive of a possible high burden of zinc deficiency. Moreover, a pilot survey completed in 2015 found that ~ 62% of children (6- 23 months) were zinc deficient, based on plasma zinc concentrations (<65 μg/dL) [23]. Under-five mortality rate in Laos is estimated at 71 per 1000 live births and the incidence of acute diarrheal illnesses is estimated at 894 per 10000 [25]. At the time of the study, the province had no existing programs to reduce the risk of micronutrient deficiencies or treat diarrhea with therapeutic zinc supplementation.

The study area ( ~ 5300 km^2^) included 300 rural villages from five districts (Nongbok, Xebangfai, Mahaxay, Xaibuathong and Yommalat). All households with potentially eligible children were invited for screening. In Nongbok district, which was relatively more accessible compared to the other districts, the invitation for screening and subsequent enrollment were restricted only to villages in the catchment areas of health centers where the overall stunting prevalence among children 6-23 months was ≥25% [23].

### Sample size considerations

To detect an effect size of 0.2 SD in mean diarrhea incidence between any two groups, with 90% power and 5% type-1 error rate, a total sample size of 710 children per group was required. The effect size of 0.2 SD was based on results of previous meta-analyses, which reported a 20% reduction in diarrhea incidence among children receiving preventive zinc supplements [2,16,26]. Allowing for 15% attrition, a total sample of 835 children (rounded up to 850) per group was proposed, and 3400 children were targeted for enrollment.

### Inclusion and exclusion criteria

Children were considered eligible to participate if they met the following criteria: 6-23 months of age, family intended to stay in the study area for the duration of the study, willingness to accept home visits, and at least one of the caregivers (mother, father, legal guardian) provided written informed consent. Children were excluded from participation if they presented with any of the following health conditions: severe anemia (hemoglobin <70 g/L), severe wasting (weight-for-length z-score (WLZ)<-3 SD with respect to the WHO 2006 standards [27]), bipedal edema, severe illness warranting hospital referral, congenital abnormalities possibly interfering with growth, chronic medical conditions requiring frequent medical attention, known HIV infection of the index child or the child’s mother, ongoing use of micronutrient supplements, or current participation in another research study.

### Randomization and study interventions

A statistician at the University of California Davis randomly assigned study ID numbers to the four study arms, using a block randomization scheme with block lengths of four or eight. This predetermined randomization list was used during enrollment. In the event that multiple eligible siblings resided in the same household, only the youngest was enrolled. Twins were randomized as one unit (ie, given the same intervention), but only one was randomly selected for inclusion at the time of data analyses.

**Figure 1:**
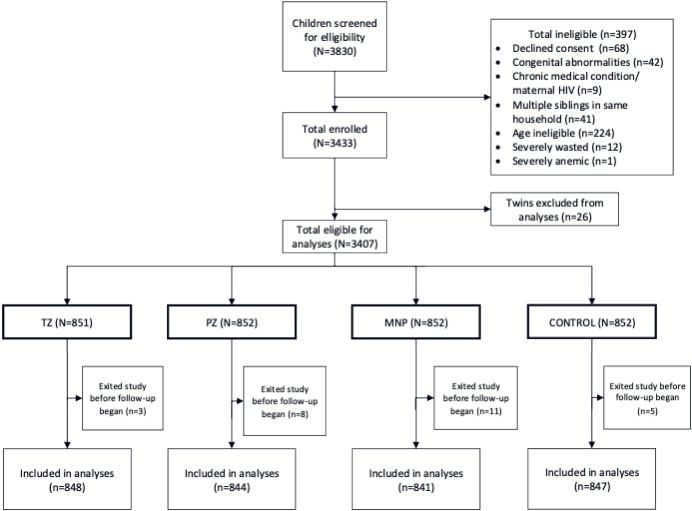
Flow diagram of study participant randomization and inclusion into data analyses. MNP – Micronutrient powder, PZ – preventive zinc, TZ – therapeutic zinc.

Children were individually randomized into one of four study groups (**Table 1** and **Figure 1**) as follows: 1) the therapeutic zinc (TZ) supplementation group received a daily placebo preventive supplement tablets and a therapeutic zinc tablet containing 20 mg each day for 10 days for diarrhea treatment; 2) the preventive zinc (PZ) supplementation group received a daily preventive zinc supplement tablet containing 7 mg zinc and placebo therapeutic tablets for diarrhea treatment; 3) the MNP group received a daily preventive MNP containing 10 mg zinc, 6 mg iron and 13 other micronutrients (**Table 1**) and placebo therapeutic tablets for diarrhea treatment; or 4) the placebo control group received daily placebo powder and placebo therapeutic tablets for diarrhea treatment. Thus, each child, regardless of the group allocation received both a preventive and a therapeutic supplement. The therapeutic intervention for diarrhea treatment was delivered as a “diarrhea kit”, containing a 10-tablet blister pack of assigned therapeutic supplements, 3 ORS packages and illustrative instructions. Caregivers were instructed to initiate diarrhea treatment as soon as a child had ≥3 liquid or semi-liquid stools per day. Specifically, caregivers were instructed to provide ORS while the diarrhea episode lasted and to give one therapeutic tablet per day for 10 days. In addition, children were to continue taking the assigned preventive supplement during diarrhea episodes. When appropriate at the time of the weekly home visit, the field workers reminded the caregiver to initiate and/or continue diarrhea treatment until the 10-day regimen was completed. After the completion of a therapeutic regimen, caregivers were given a new diarrhea kit corresponding to the assigned study group and were instructed to use it during the next diarrhea episode. In the event of persistent diarrhea (ie, diarrhea lasting more than 14 days), the therapeutic tablets were replaced with ZincFant (Nutriset SAS, Malaunay France), non-blinded tablets containing 20 mg of zinc, which the caregiver was instructed to give to the child for the next 10 days. Caregivers where also instructed to temporarily stop providing the assigned study interventions to the child until the “ZincFant” course was completed. The preventive and therapeutic zinc and placebo tablets were produced by Nutriset SAS (Malaunay, France). The MNP and placebo powder sachets were produced by DSM Fortitech Asia Pacific (Banting, Malaysia). Supplements were pre-labelled (by the manufacturer) with four different numerical codes, along with the letters P (for preventive supplements) or T (for therapeutic supplements). In addition, intervention products were assigned specific colors (one color per intervention group) to facilitate correct delivery and tracking.

**Table 1:** Preventive and therapeutic supplements provided to each study group in the Lao Zinc Study

	Center TZ	Center PZ	Center MNP	Center CONTROL
Preventive supplement	Placebo tablet	Zn-containing tablet (7 mg/day)	MNP (containing 10 mg Zn, 6 mg Fe, +13 other micronutrients*)	Placebo powder
Therapeutic supplement for diarrhea	ORS + Zn tablet (20 mg/day) for 10 day starting with acute diarrhea episodes	ORS + Placebo tablet	ORS + Placebo tablet	ORS + Placebo tablet

MNP – micronutrient powder; ORS – oral rehydration salt; PZ – preventive zinc supplementation; TZ – therapeutic zinc supplementation for diarrhea

*MN contents of one daily dose MNP sachet: vitamin A (400 μg RE), thiamin (0.5 mg), riboflavin (0.5 mg), niacin (6 mg), vitamin B6 (0.5 mg), folic acid (150 μg DFE), cyanocobalamin (0.9 μg), ascorbic acid (30 mg), cholecalciferol (5 mg), dl-α-tocopheryl acetate (5 mg TE), copper sulphate anhydrous (to provide 0.56 mg copper), potassium iodate (to provide 90 μg iodine), ferrous fumarate (to provide 6 mg iron), selenium selenite (to provide 17 μg selenium), zinc gluconate (to provide 10 mg zinc).

At enrollment, caregivers were instructed on how to administer the study products to their child, and field workers repeated these instructions once per month during home visits. For both the preventive and therapeutic dispersible zinc and placebo tablets, caregivers were instructed to dissolve one tablet with clean water or breastmilk and feed the resulting suspension to the child at least 30 minutes before or after a meal. Caregivers were advised to mix the entire contents of a MNP or placebo powder sachet into a small amount of semi-solid food that the child could easily consume. Each child was visited weekly for 32-40 weeks, unless lost to follow-up. Supplements and ORS were replenished during these weekly home visits. During the weekly visits, caregivers were interviewed to record reported consumption of the intervention products, and unused blister packages and sachets were collected to assess adherence.

### Data collection and consent procedures

On the day of enrollment, consent materials were presented first in a group education session conducted by a medical doctor, followed by a one-on-one session with a study nurse. Caregivers who wished to participate in the study were asked to sign (or fingerprint) the consent statement in the presence of an independent observer. Children with written, informed consent by one parent or a legal caregiver were subsequently screened for eligibility. At baseline, mid-point (after ~ 18 weeks) and endline (after 32-40 weeks), duplicate anthropometric assessments were recorded by trained and standardized anthropometric teams. Measurements included weight to the nearest 0.02 kg (SECA 383, Seca, Hamburg, Germany), recumbent length to the nearest 0.1 cm (SECA 416) and mid-upper arm circumference (MUAC) to the nearest 0.1 cm (ShorrTape©, Measuring Tape, Weigh and Measure, Olney, MD, USA). Maternal weight was measured to 50 g precision (SECA 874) and maternal height to 0.1 cm precision (SECA 213) at baseline or at another time over the course of the study.

Anemia status, based on capillary hemoglobin concentrations, was assessed for all children at baseline and endline (32-40 weeks later) by Hemocue® Hb 301 System (Hemocue AB, Angelholm, Sweden). To assess zinc status and inflammation, a total of 760 children (190 per group) were targeted for blood draw. Due to logistical challenges of the large study area, blood had to be drawn from the children enrolled in the health districts closest to provincial capital [23]. Among these children, approximately 7 ml of venous blood were successfully collected from 701 of these children by trained nurses into evacuated, trace element-free 7.5 ml Lithium-Heparin tubes (Sarstedt AG & Co, Numbrecht, Germany). The heparinized samples were maintained at 4-8°C in portable cooler boxes until they were transported to field laboratories later the same day for processing. In the field laboratory, the heparinized blood samples were centrifuged (PowerSpin Centrifuge Model LX C856; United Products & Instruments, Inc., Dayton, NJ) at 1097 × g (3100 RPM) for 10 minutes and the plasma was aliquoted. Plasma samples were stored in the field laboratory at -20°C, and later shipped on dry ice to collaborating laboratories for analyses. Samples from a subgroup of 575 children with baseline and endline plasma sample were randomly selected for laboratory analyses.

### Weekly morbidity surveillance

All illness-related symptoms, including the reported number of liquid or semi-liquid stools per day, reported and confirmed fever, signs and symptoms of respiratory tract infections, hospitalizations and clinic visits, and any consumption of medications were assessed during weekly home visits by trained morbidity surveillance workers. Caregivers were given a paper-based chart weekly to document daily morbidity symptoms and supplement consumption. While administering the electronic questionnaire, the field worker would refer to the chart as needed to ensure consistency or to clarify disparities between the information on the chart and the responses provided by the caregiver. Axillary body temperature was measured when fever was reported in the previous 24 hours and once per month independent of any reported fever.

### Laboratory analyses

Plasma zinc was analyzed by inductively coupled plasma optical emission spectrophotometry (ICP-OES Agilent 5100 SVDV, Santa Clara, CA, USA) at the Children’s Hospital of Oakland Research Institute (CHORI, Oakland, CA, USA) [28]. Inflammatory markers (CRP and AGP) were measured using a sandwich enzyme-linked immunosorbent assay (ELISA) technique at the VitMin Laboratory (Willstaett, Germany) [29].

### Data entry and analysis

All data were recorded electronically via a customized CommCare-HQ (Dimagi, Boston, USA) application deployed on portable Samsung tablets (Samsung Galaxy, Tab-4, Suwon, South Korea). A statistical analysis plan was published prior to analyses [30] and strictly followed to minimize bias. The groups’ identities were revealed only after the data analyses were completed and the study investigators reached consensus on the interpretation of results. All analyses were done with STATA statistical software, release 13 (StataCorp, Austin, TX, USA) and SAS version 9.4 (SAS Institute, Cary, NC, USA).

### Definition of outcomes and indicators of child health

We defined diarrheal illness as 3 or more liquid or semi-liquid stools in a 24-hour period. A diarrhea episode was defined as all contiguous days of diarrheal illness followed by at least 2 diarrhea-free days. Acute lower respiratory infection (ALRI) was defined as any episode in which the caregiver reported severe or constant cough with respiratory difficulty (eg, wheezing/stridor, chest in-drawing or difficulty breathing); an ALRI episode was defined as an ALRI illness followed by at least 3 days free of ALRI. Acute upper respiratory infection (AURI) was defined as any episode in which the caregiver reported cough and a purulent nasal discharge; an AURI episode was defined as an AURI illness followed by at least 7 days free of AURI. Additional morbidity outcomes of interest included reported fever or confirmed fever (axillary temperature >37.5°C), severe diarrhea (defined as ≥6 liquid or semi-liquid stools in a 24h period) and dysentery (ie, diarrhea with blood in stool). We assessed treatment effects on incidence density (defined as the number of episodes divided by number of days at risk), longitudinal prevalence (defined as the proportion of days with illness) and duration (defined as the number of days per episode) of diarrhea, ALRI and AURI.

Weight-for-age z-scores (WAZ), length-for-age z-scores (LAZ) and WLZ were used to define growth status, and stunting (LAZ<-2 SD), underweight (WAZ<-2 SD) and wasting (WLZ<-2 SD) were based on the WHO growth standards [31].

Adherence to the daily preventive supplement was defined as the total number of days in which the preventive supplement was consumed, expressed as a fraction of the total days of observation per child. We defined total intake of therapeutic tablets as the total number of days the supplement was reportedly given to the child. In addition, we also assessed the total number of tablets used in treating diarrhea episodes (ie, tablets given within 10 days of illness), the number of episodes treated (defined as treatment initiated within 7 days of diagnosis) and the number of tablets given within 10 days of treatment initiation.

### Statistical analyses

All analyses were done on a complete-case, intention-to-treat basis. In all analyses, the intervention group was considered the primary exposure variable. Negative binomial models with an offset were used to assess group differences in incidence density, longitudinal prevalence and duration of diarrhea, ALRI and AURI. For incidence density, we modelled the total count per child with the days at risk as the offset. For longitudinal prevalence, we modelled the total number of disease-specific sick days with the total days of observation as the offset and for disease-specific duration, the total number of disease-specific sick days was modelled with the total number of episodes as the offset. Cox proportional-hazard models were used to assess time to diarrhea events. Models first assessed a global difference in intervention effect using a likelihood ratio test, and post-hoc pairwise differences were assessed subsequently in the event of a statistically significant global difference (global *P*-value <0.05). In all cases of statistically significant pair-wise comparisons, multiple hypothesis testing adjustments were made to determine the sensitivity of estimates. All models were rerun to assess evidence of potential effect modification by baseline variables, including age (at enrollment), physical growth status, sex, socioeconomic status, complementary feeding practices, breastfeeding status, maternal literacy and other indicators, by incorporating an interaction term. For each outcome, intervention effect parameters were estimated using models that controlled for age at enrollment, sex and district as the only covariates. Full lists of the other covariates and effect modifiers explored are available in the published statistical analysis plan [30]. In the event of significant interactions (*P* < 0.1), marginal effect plots were developed to visually display the modifying effects and stratified analyses using various categories were performed to better understand the nature of the effect modification.

Because of the nature of the therapeutic zinc intervention, we also performed secondary analyses to explore the prophylactic effects of therapeutic zinc supplementation on subsequent diarrhea incidence. Since any effects of therapeutic zinc are limited to the period after initiation of therapeutic supplementation, we reran the main models after excluding children who never consumed the therapeutic supplements and all observation days prior to initiation of therapeutic supplementation among children in all 4 groups. Additional exploratory analyses were done to assess treatment impact on the time to first and second diarrhea episodes.

## RESULTS

From 3830 children screened, 3407 eligible children were enrolled and individually randomized into the TZ (n = 851), PZ (n = 852), MNP (n = 852) and Control (n = 852) groups (**Figure 1**). Approximately 13% of study participants, including 10% in the TZ group, 13% in PZ and Control groups, and 17% in the MNP group (*P* = 0.01) dropped out before the scheduled endline. We excluded from the analyses children (n = 26) who exited the study immediately after enrollment and therefore did not provide morbidity data. As previously reported, the characteristics of the children lost to follow-up were statistically similar to those who completed the study with respect to baseline age, maternal variables and anemia [32].

The mean age of participating children was 14.3 ± 5.0 months at baseline (**Table 2**). At enrollment, the prevalence of stunting and anemia were 39.6% and 55.1%, respectively; and 75.4% had low plasma zinc concentration. About 73% of children were still breastfeeding at baseline.

**Table 2:** Baseline characteristics among children and mothers included in the analyses

	All N = 3380	TZ N = 848	PZ N = 844	MNP N = 841	Control N = 847
Child characteristics*					
Age (months)	14.3 ± 5.0	14.4 ± 5.2	14.2 ± 5.1	14.3 ± 5.0	14.1 ± 5.1
Males, n (%)	1503 (51.1)	392 (51.3)	370 (50.1)	356 (50.8)	385 (52.0)
LAZ	-1.75 ± 1.08	-1.79 ± 1.11	-1.81 ± 1.03	-1.74 ± 1.07	-1.68 ± 1.09
Stunting, n (%)	1328 (39.3)	330 (38.9)	348 (41.3)	339 (40.3)	311 (36.7)
Hemoglobin, (g/L)	107.0 ± 1.1	107.7 ± 1.1	107.7 ± 1.1	107.4 ± 1.1	107.8 ± 1.0
Anemia, n (%)	1871 (55.4)	468 (55.2)	466 (55.2)	476 (56.6)	461 (54.3)
**Zinc Status (n = 568):†**					
Plasma zinc (μg/dL)	54.2 (14.2)	54.4 (13.0)	52.2 (16.6)	55.2 (13.8)	555,1(13.5)
Zinc deficiency, n (%)	432 (75.4)	108 (74.5)	110 (74.3)	101 (72.1)	113 (80.7)
**Maternal:^a^**					
Maternal age (years)	28.0 ± 5.9	26.7 ± 6.0	27.8 ± 5.7	28.1 ± 5.9	27.0 ± 6.0
Maternal BMI (kg/m^2^)	21.4 ± 2.9	21.4 ± 2.9	21.4 ± 3.0	21.5 ± 2.7	21.4 ± 3.0

TZ – therapeutic zinc, PZ – preventive zinc, MNP – micronutrient powder, LAZ – length for age z-scores, BMI – body mass index

*Values represent mean±SD for continuous variable or N (%) for dichotomized variables. Stunting defined as LAZ<-2; anemia defined as Hb <110 g/L.

†Values represent median (IQR) for continuous variable or N (%) for dichotomized variables. Zinc deficiency defined as plasma zinc concentration <65μg/dL. Plasma zinc data adjusted for inflammation.

Reported adherence to the daily preventive supplements was 92% (**Table 3**). Over the course of the follow-up period, 2013 children (representing ~ 60% of the study population) were diagnosed with at least one diarrhea episode; among those children about 97% received the therapeutic tablet at least one time. On days that children consumed the therapeutic supplement, they concurrently consumed the preventive supplement ~ 92% of the time. Approximately 87% of all diarrhea episodes were treated (ie, treatment initiated within 7 days of diarrhea onset) across all groups (*P* = 0.90 for group-wise comparison, **Table 3**). The total number of children who never had diarrhea were 342 (TZ), 350 (PZ), 368 (MNP) and 333 (Control). On average, children consumed 7 out of the 10 required tablets per diarrhea episode, with no differences between groups. Of the treated episodes (ie, episodes in which zinc therapy was initiated within 7 days after illness onset), 76% (in TZ and PZ), 78% (in MNP) and 77% (in Control) of tablets consumed were given within 10 days of the initiation of treatment. Based on reported adherence to both the preventive and therapeutic supplements, children in the PZ group received an average of ~ 6.5 mg zinc/d, those in the MNP group ~ 9.0 mg zinc/d and those in the TZ group ~ 0.8 mg zinc/d over the study duration (**Table 3**). ORS was given ~ 1 day per episode, with no differences between groups (*P* = 0.95).

**Table 3:** Adherence to study interventions based on weekly caregiver reports

Adherence to supplementation*	TZ	PZ	MNP	CONTROL	*P* value
**N children**	848	844	841	847	
**Preventive†**					
Total preventive tablets/sachets used, n (%)	183 762 (92.4)	179 299 (92.1)	166 009 (89.2)	178 116 (91.0)	<0.001
**Therapeutic**					
Total therapeutic tablets given, n	8124	7972	7449	7962	0.46
Total number of diarrhea episodes reported	1128	1095	1091	1157	
Days therapeutic tablet given per episode‡	6.8 ± 4.3	6.6 ± 3.9	6.2 ± 4.0	6.8 ± 4.7	0.10
Episodes treated (%)§	982 (87)	949 (87)	953 (87)	999 (86)	0.902
Tablet given within 10 days of initiation of treatment, n (%)	6177 (76)	6080 (76)	5842 (78)	6188 (77)	<0.01
Tablets given per treated episode	6.2 ± 3.0	6.2 ± 3.1	5.9 ± 3.3	6.2 ± 3.0	0.22
Days ORS used	2487	2705	2618	2651	0.47
Days ORS used per episode	0.64 ± 0.7	0.62 ± 0.7	0.63 ± 0.7	0.65 ± 0.7	0.95
Days treated with therapeutic tablet among children who never had diarrhea	787	804	717	732	0.73

TZ – therapeutic zinc, PZ – preventive zinc, MNP *–*micronutrient powder, ORS – oral rehydration salt

*Excluded days for which the status of supplement intake was unknown.

†Adherence to preventive supplement defined as percentage of total observation days for which daily preventive supplement was given to the child.

‡The “days therapeutic tablet given per episode” include the total number of days the child took the therapeutic tablet regardless of whether or not is was given in relation to a diarrhea episode.

§A treat episode is defined as initiation of treatment (ie, consumption of the first tablet) within 7 days of the onset of diarrhea.

**Figure 2:**
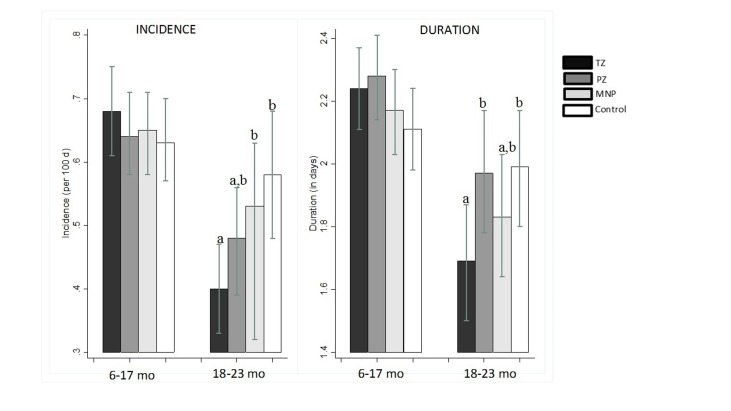
Modifying effect of child age on the responses to therapeutic zinc supplementation for diarrhea, daily preventive zinc supplementation, daily MNP and placebo on diarrhea incidence and duration among young Laotian children. TZ – therapeutic zinc, PZ – preventive zinc, MNP *–* micronutrient powder. All values adjusted for age, sex, and district. Values with different superscripts are significantly different (*P* < 0.01).

Overall, acute diarrhea incidence was low ( ~ 0.6 episodes per 100 days at risk), and did not differ among the four intervention groups (*P* = 0.90, **Table 4**). On average, a diarrhea episode lasted for about 2 days, with no group-wise differences in diarrhea duration (*P* = 0.40). However, we found a modifying effect of age on the group-specific incidence (*P* for interaction = 0.06), duration (*P* for interaction = 0.01) and longitudinal prevalence of diarrhea (*P* for interaction = 0.01). Specifically, there was a treatment effect in older children (>18 months at baseline), but not in the younger children. In the subset of children older than 18 months, the diarrhea incidence in the TZ group (0.40 per 100 days at risk) was significantly lower (*P* < 0.01) than in both the MNP (0.54 per 100 days at risk, *P* = 0.035) and Control (0.58 per 100 days at risk, *P* = 0.004) groups, but did not differ from the PZ group (0.47 per 100 days at risk, *P* = 0.191). Similarly in children older than 18 months, but not younger ones (**Figure 2**), the average duration of diarrhea in the TZ group (1.7 ± 0.06 days) was significantly shorter than among children in the Control (2.0 ± 0.1, *P* = 0.03) and PZ groups (2.0 ± 0.1, *P* = 0.03). No other significant pairwise group differences in diarrhea incidence and duration were identified. The age-specific effects on diarrhea incidence and duration translated into similar effects with respect to the longitudinal diarrhea prevalence (Table S1 in the **Online Supplementary Document**). In particular, the longitudinal prevalence of diarrhea among children >18 months was lower in the TZ group (0.74 days per 100 days at risk) compared to the PZ (1.12 days per 100 days at risk, *P* = 0.02), the MNP (0.99 days per 100 days at risk, *P* = 0.065) and the Control groups (1.26 days per 100 days at risk, *P* = 0.002).

**Table 4:** Effects of providing therapeutic zinc supplements for diarrhea or daily preventive zinc supplements or MNP on diarrhea and acute respiratory infections among young Laotian children*

	TZ	PZ	MNP	Control	Global *P*-value	*P*-value† for age interaction
N children	848	844	841	847		
Total observation days	198 975	194 603	186 133	195 804		
**Primary diarrhea outcomes:**						
Total number of diarrhea episodes	1128	1095	1091	1157		
Diarrhea incidence (episodes per 100 days at risk)	0.60 ± 0.03	0.60 ± 0.03	0.62 ± 0.03	0.62 ± 0.03	0.90	0.06
Total number of days with diarrhea	2477	2443	2287	2470		
Longitudinal prevalence (days with diarrhea per 100 days observed)	1.48 ± 0.09	1.53 ± 0.09	1.42 ± 0.09	1.44 ± 0.09	0.82	0.01
Average duration (days per episode)	2.10 ± 0.06	2.20 ± 0.06	2.08 ± 0.06	2.08 ± 0.09	0.40	0.01
**Secondary diarrhea outcomes:**						
Incidence of severe diarrhea episodes	0.08 ± 0.01	0.09 ± 0.01	0.08 ± 0.01	0.07 ± 0.01	0.50	0.55
Incidence of dysentery	0.04 ± 0.01	0.04 ± 0.01	0.03 ± 0.01	0.04 ± 0.01	0.57	0.53
**Acute lower respiratory tract infection (ALRI**):						
Total number of ALRI episodes	17	7	11	7		
Total number of days with ALRI	38	25	27	23		
Incidence (episodes per 100 d at risk)	0.009 ± 0.002	0.004 ± 0.001	0.006 ± 0.002	0.004 ± 0.001	0.14	0.08
Longitudinal prevalence (days with ALRI per 100 days observed)	0.027 ± 0.013	0.012 ± 0.006	0.015 ± 0.007	0.007 ± 0.004	0.49	0.16
Average duration (days per episode)	2.40 ± 0.41	3.45 ± 0.76	2.35 ± 0.47	2.14 ± 0.67	0.50	0.87
**Acute upper respiratory tract infection (AURI)**‡:						
Total number of AURI episodes	167	178	155	187		
Total number of days with AURI	709	782	667	830		
Incidence (episodes per 100 d at risk)	0.09 ± 0.11	0.10 ± 0.11	0.09 ± 0.11	0.10 ± 0.11	0.72	0.60
Longitudinal prevalence (days with AURI per 100 days observed)	0.32 ± 0.06	0.46 ± 0.9	0.34 ± 0.06	0.41 ± 0.07	0.45	0.98
Average duration (days per episode)	4.17 ± 0.26	4.17 ± 0.25	4.27 ± 0.26	4.17 ± 0.24	0.99	0.52

MNP – micronutrient powder, PZ – preventive zinc, TZ – therapeutic zinc

*All models adjusted for age, sex, district and respective baseline values.

†*P* value statistical significance of interaction with continuous age at enrolment measured in months. No other effect modification were observed.

‡Acute lower respiratory tract infection (ALRI) defined as constant or severe cough with either wheezing, stridor, or chest in-drawing. Acute upper respiratory tract infection (AURI) defined as cough with nasal discharge; Global *P*-value represents statistical significance of main effect.

In secondary analyses, in which observation days occurring prior to the first treatment were excluded from analyses in all four intervention groups, TZ significantly reduced the subsequent incidence of diarrhea relative to the Control group (*P* < 0.01) among children >18 months, but not in the younger children (Table S2 in the **Online Supplementary Document**).There were no treatment effects on dysentery or severe diarrhea episodes (**Table 4**). There was no evidence that any of the interventions significantly delayed the time to first or subsequent diarrhea episodes (data not shown).

The incidence of ALRI was low, ranging from 0.004 to 0.009 episodes per 100 days at risk across the four groups, with no differences among groups (*PP =* 0.14; **Table 4**). The incidence of AURI was also low (average of ~ 0.095 episodes per 100 days at risk) with no group-wise differences (*P* = 0.72). We observed a significant age interaction regarding the study group effects on ALRI (*P* for interaction = 0.08). However, the total number of ALRI events was too small (n = 42 ALRI episodes for the entire study duration) to adequately model the age-specific trends.

## DISCUSSION

In the present trial, the provision of 20 mg zinc for diarrhea treatment reduced the duration of the diarrhea episodes and the incidence of future episodes in older children, but had no significant effects among younger children. Daily preventive zinc supplements, delivered either as single-nutrient tablets, or as part of MNP, had no impact on either the incidence or duration of diarrhea. There were no overall treatment effects on ALRI or AURI in any of the intervention groups. The MNP, containing a low iron dose of 6 mg, was not associated with an overall increase in diarrhea, ALRI, AURI or any other adverse events.

The WHO and UNICEF recommend the provision of 20 mg zinc as part of diarrhea management [7,33], and meta-analyses have consistently found a beneficial impact of therapeutic zinc supplementation on reducing diarrheal duration [5,34]. Specifically, in children 6 months and older, diarrhea treatment with zinc has been shown to shorten the duration of acute episodes by approximately half a day, and by about 1 day among children showing signs of malnutrition [35]. Thus, the limited impact of TZ on diarrhea duration ( ~ 0.3 days reduction among TZ compared to Control and PZ), which was found only among children >18 months of age in the present study, was unexpected. This observation may be due in part to the mild degree of diarrhea severity observed overall in the study population. In particular, the duration of diarrhea observed in this study was considerably shorter ( ~ 2 days/episode) than the 3-5 days typically seen across studies that previously reported a beneficial effect of therapeutic zinc supplementation regimens [35].

Thus far, the evidence seems to suggest that therapeutic zinc is only effective in reducing diarrhea duration in older infants and children [36-39]. Our observation of an impact in older children, but not younger, children is consistent with this pattern. This observation may reflect age-dependent maturation of the zinc-reactive immune responses to diarrhea or possible protection of more intensive breast feeding among the younger children [40]. Because the strength of adaptive immune response generally increase with repeated exposure, it is plausible that the zinc may have elicited a stronger immune response in the older children [40].

There is little information available on the effect of TZ on the incidence of subsequent episodes of diarrhea. Our results are consistent with the study by Baqui et al, which found a 15% reduction in the subsequent incidence of diarrhea in Bangladeshi children following diarrhea treatment with 20 mg/d zinc for 14 days [6]. On the other hand, these results differ from those of a study in Kenyan children, which found no impact of adjunctive zinc therapy (10 mg/d for 10 days during diarrhea episodes) on the incidence of subsequent diarrhea episodes [41]. A likely reason for the prophylactic benefits observed in this population is that recurring diarrhea episodes closely followed the prior episode. For instance, among children in the placebo group who had >1 diarrhea episode, roughly 60% of the successive episodes occurred within 3 months of the prior episodes, which is within the 2- to 3-month window during which a prophylactic effect may be conferred by the zinc therapy [6].

Meta-analyses of preventive zinc supplementation trials have consistently shown a reduction in diarrhea incidence [2,42]. The lack of effect in the PZ in the current study was surprising, especially considering the high prevalence of zinc deficiency at baseline (>70%). Compared with previous trials conducted in Asia, our results are inconsistent with a trial conducted in Bangladesh [43], which found a small reduction in diarrhea incidence in the zinc supplemented group. On the other hand, our results are consistent with a results from a large trial by Tielsch et al [44], which found no impact of daily preventive zinc supplementation on the incidence of diarrhea and respiratory infections in 1 to 35 month-old Nepalese children, despite using a higher 10 mg/d dose of zinc for preventive supplementation in a setting with a relatively higher diarrhea burden. The differences in impact may be explained by a number of factors, including the dose of zinc used, the underlying zinc status of the population, the prevailing risk of diarrhea, the age of the study population and possible confounding effects of child feeding practices.

The equivalent daily dose of zinc used in previous trials ranged from ~ 1 mg to 21.4 mg zinc/d, delivered either daily or intermittently in amounts ranging from 1-70 mg [2]. We chose a preventive dose of 7 mg zinc per day for the present study because an earlier dose-response study by Wuehler et al found that the greatest impact on diarrhea occurred with a dose of 6-7 mg zinc/d [45]. Considering that the therapeutic use of 20 mg zinc in this study was associated with a reduction in diarrhea incidence among older children, it is possible that a higher dose may have been needed in this population. Alternatvely, it is possible that other factors than the dose of zinc were responsible for the observed results, as proposed above.

Meta-analyses of previous studies also concluded that preventive zinc supplementation reduced the incidence of ALRI by 13%-15% [2,42]. A possible explanation for the failure to detect an effect of preventive zinc supplementation in this study is the methods used to identify ALRI. The previous meta-analyses concluded that the impact on ALRI was detectable only when more specific case definitions were applied, such as physician-assigned or radiologically-based diagnosis, whereas no impact was found when the case definition was based on parental recall, as was done in the present study [2,42]. In this study, ALRI and AURI were diagnosed by caregiver recall, which has been shown to be unreliable in some contexts [46]. However, a further review of the reported ALRI cases showed that most were accompanied by other indicators of severe disease, including hospital admissions and elevated body temperature, and perhaps suggesting that the children may have experienced respiratory distress. The prior meta-analyses of preventive zinc supplementation have also concluded that co-administration of zinc with other micronutrients, including iron and vitamin A, may reduce or eliminate the impact zinc supplementation on health outcomes [2,16]. Our results in the MNP group are consistent with this observation. Despite increasing the zinc dose to 10 mg (from the typical 4.1-5 mg/sachet found in conventional formulations), the MNP did not have an impact on diarrhea, ALRI and AURI. These results are also consistent with a study by McDonald et al which found no effect of a multiple micronutrient supplementation on diarrhea incidence either [47]. Notably, the latter study used a capsule containing 5 mg zinc along with 6 other vitamins, but not iron. Thus, the lack of effect of MNP on morbidity prevention may also be explained by the effects of other MNs on the efficacy of zinc supplementation [48].

Our results differ from several previous MNP trials, which found an increased risk of infections [20,49]. In Pakistani children, Soofi et al reported that daily MNP supplementation was associated with an increased duration of all acute diarrhea as well as bloody diarrhea specifically [20,49]; and in Ghana Zlotkin et al found a higher rate of hospitalization among children who received MNP, mostly due to diarrhea and respiratory infections [21]. Both of these studies used an MNP formulation containing 12.5 mg iron per sachet, as opposed to the 6 mg used in the present trial. Furthermore, there is evidence that iron-containing MNP adversely affects the gut microbiome by decreasing the abundance of non-pathogenic commensal bacterial such as *bifidobacteria*, while increasing the abundance of pathogenic *E coli* [50-52]. We did not assess the gut microbiome in this trial, but if such changes had occurred in the microbiome of our study population, this did not result in an increased diarrhea incidence. It is worth mentioning that in a secondary analyses of modifying effects, the MNP was associated with a small, marginally significant increase in diarrhea among the children with inherited hemoglobin disorders [53]. The presence of hemoglobin variants may disrupt iron utilization [54], but it is not clear why MNP might increase diarrhea among children with inherited hemoglobin disorders.

Of the plausible explanations for the lack of main effects in this trial, the most likely is perhaps the low incidence of diarrhea observed in this population. It is estimated that on average, children in settings similar to our study population experience ~ 3 episodes of diarrhea episodes per year [5]. In this trial, children experienced the equivalence of ~ 2 episodes per child-year, assuming uniform transmission intensity throughout the year. The low burden of diarrhea may also explain the lack of effect on duration (by the TZ). The duration of diarrhea observed in this study ( ~ 2 days/episode) was shorter than the 3-5 days typically seen across studies that found an effect with ≤20 mg/d zinc regimen [35]. It is unlikely that the lack of effect is attributable to non-adherence. In the present trial, reported daily adherence to the preventive supplements was high ( ~ 91% overall), and although the reliability of caregiver reports remain debatable [55], the high adherence reported in this study was confirmed by an increase in plasma zinc concentrations in the PZ group and increased concentrations of plasma ferritin and soluble transferrin receptor in the MNP group [32].

The present study has several strengths that support the reliability of the results presented in this paper. First, we implemented the study in a population that had a high prevalence of zinc deficiency at baseline (>70%), and therefore a presumably greater potential for impact. In addition, we used an individual-level randomization design, and we implemented an active disease surveillance protocol whereby participating families were visited weekly for up to 9 months. We also implemented a rigorous set of masking procedures throughout the field implementation and data analyses, minimizing potential investigator-related biases. A potential threat to this blinding protocol is the preventive supplement type. Dispersible tablets were used for the preventive supplements in the PZ and TZ groups, and powder sachets for the MNP and Control groups. However, because there were two groups of each type of preventive supplement (ie, tablet or powder), it was not possible to identify the allocation of the individual study group.

## CONCLUSIONS

In conclusion, the present study provides some support for the use of the therapeutic (but not preventive) zinc supplementation to reduce the diarrhea burden in older children. However, because TZ was only beneficial in the older children, other interventions, such as the promotion of adequate breastfeeding, safe complementary feeding and appropriate hygiene and sanitation practices, are still needed to prevent diarrhea among infants and young children in this and similar populations. The lack of impact of PZ on morbidity and growth [32], despite improving plasma zinc status, implies that daily provision of zinc supplements may not be beneficial to achieve these functional outcomes in this population. MNP neither showed any benefits nor any adverse effects on the diarrhea burden of young children in the present study; but, as reported previously, had a positive impact on the micronutrient status [32].

## Additional material

Online Supplementary Document

## References

[1] Walker CL Rudan I Liu L Nair H Theodoratou E Bhutta ZA Global burden of childhood pneumonia and diarrhoea. Lancet. 2013;381:1405-16. 10.1016/S0140-6736(13)60222-6 23582727 PMC7159282

[2] Brown KH Peerson JM Baker SK Hess SY Preventive zinc supplementation among infants, preschoolers, and older prepubertal children. Food Nutr Bull. 2009;30:S12-40. 10.1177/15648265090301S103 19472600

[3] Fischer Walker CL Aboubaker S Van de Weerdt R Black RE Zinc for diarrhoea management in Sub-Saharan Africa: a review. East Afr Med J. 2007;84:441-9. 18074963 10.4314/eamj.v84i9.9554

[4] Wessells KR Singh GM Brown KH Estimating the global prevalence of inadequate zinc intake from national food balance sheets: effects of methodological assumptions. PLoS One. 2012;7:e50565. 10.1371/journal.pone.0050565 23209781 PMC3510064

[5] Lazzerini M Wanzira H Oral zinc for treating diarrhoea in children. Cochrane Database Syst Rev. 2016;12:CD005436. 10.1002/14651858.CD005436.pub5 27996088 PMC5450879

[6] Baqui AH Black RE El Arifeen S Yunus M Chakraborty J Ahmed S Effect of zinc supplementation started during diarrhoea on morbidity and mortality in Bangladeshi children: community randomised trial. BMJ. 2002;325:1059. 10.1136/bmj.325.7372.1059 12424162 PMC131175

[7] World Health Organization. UNICEF. Clinical management of acute diarrhoea in children: WHO/UNICEF joint statement. Geneva: World Health Organization; 2004. Available: http://wwwwhoint/maternal_child_adolescent/documents/who_fch_cah_04_7/en/. Accessed:September, 13, 2017.

[8] World Health Organization. UNICEF.: Zinc supplementation in the management of diarrhoea. http://wwwwhoint/elena/titles/zinc_diarrhoea/en/ 2016, Accessed: 28 September 2016.

[9] Ram PK Choi M Blum LS Wamae AW Mintz ED Bartlett AV Declines in case management of diarrhoea among children less than five years old. Bull World Health Organ. 2008;86:E-F. 10.2471/BLT.07.041384 18368194 PMC2647400

[10] Sabot O Schroder K Yamey G Montagu D Scaling up oral rehydration salts and zinc for the treatment of diarrhoea. BMJ. 2012;344:e940. 10.1136/bmj.e940 22327358

[11] Wang L Song Y Efficacy of zinc given as an adjunct to the treatment of severe pneumonia: A meta-analysis of randomized, double-blind and placebo-controlled trials. Clin Respir J. 2018;12:857-64. 10.1111/crj.12646 28488366

[12] Yuan X Qian SY Li Z Zhang ZZ Effect of zinc supplementation on infants with severe pneumonia. World J Pediatr. 2016;12:166-9. 10.1007/s12519-015-0072-9 26684319

[13] Zen V Vinholes DB Wolff FH The effect of zinc on pneumonia in children: is it really ineffective? Am J Clin Nutr. 2010;92:997-8, author reply 998-9. 10.3945/ajcn.2010.30019 20685952

[14] Yamey G Zinc supplementation prevents diarrhoea and pneumonia. BMJ. 1999;319:1521A. 10591705 10.1136/bmj.319.7224.1521a PMC1117257

[15] Imdad A Bhutta ZA Effect of preventive zinc supplementation on linear growth in children under 5 years of age in developing countries: a meta-analysis of studies for input to the lives saved tool. BMC Public Health. 2011;11 Suppl 3:S22. 10.1186/1471-2458-11-S3-S22 21501440 PMC3231896

[16] Mayo-Wilson E Junior JA Imdad A Dean S Chan XH Chan ES Zinc supplementation for preventing mortality, morbidity, and growth failure in children aged 6 months to 12 years of age. Cochrane Database Syst Rev. 2014;CD009384. 10.1002/14651858.CD009384.pub2 24826920

[17] Brown KH Peerson JM Rivera J Allen LH Effect of supplemental zinc on the growth and serum zinc concentrations of prepubertal children: a meta-analysis of randomized controlled trials. Am J Clin Nutr. 2002;75:1062-71. 10.1093/ajcn/75.6.1062 12036814

[18] Jefferds ME Irizarry L Timmer A Tripp K UNICEF-CDC global assessment of home fortification interventions 2011: current status, new directions, and implications for policy and programmatic guidance. Food Nutr Bull. 2013;34:434-43. 10.1177/156482651303400409 24605694 PMC4547468

[19] De-Regil LM Suchdev PS Vist GE Walleser S Pena-Rosas JP Home fortification of foods with multiple micronutrient powders for health and nutrition in children under two years of age. Cochrane Database Syst Rev. 2011;CD008959. 10.1002/14651858.CD008959.pub2 21901727

[20] Soofi S Cousens S Iqbal SP Akhund T Khan J Ahmed I Effect of provision of daily zinc and iron with several micronutrients on growth and morbidity among young children in Pakistan: a cluster-randomised trial. Lancet. 2013;382:29-40. 10.1016/S0140-6736(13)60437-7 23602230

[21] Zlotkin S Newton S Aimone AM Azindow I Amenga-Etego S Tchum K Effect of iron fortification on malaria incidence in infants and young children in Ghana: a randomized trial. JAMA. 2013;310:938-47. 10.1001/jama.2013.277129 24002280

[22] Prentice AM Mendoza YA Pereira D Cerami C Wegmuller R Constable A Dietary strategies for improving iron status: balancing safety and efficacy. Nutr Rev. 2017;75:49-60. 10.1093/nutrit/nuw055 27974599 PMC5155616

[23] Wessells KR Brown KH Kounnavong S Barffour MA Hinnouho G-M Sayasone S Comparison of two forms of daily preventive zinc supplementation versus therapeutic zinc supplementation for diarrhea on young children’s physical growth and risk of infection: study design and rationale for a randomized controlled trial. BMC Nutr. 2018;4:39. 10.1186/s40795-018-0247-6 32153900 PMC7050875

[24] Ministry of Health and Lao Statistics Bureau. Lao People's Democratic Republic Special, 2011–12 - Lao Social Indicator Survey (MICS/DHS) Final Report (English) Vientiane: Ministry of Health and Lao Statistics Bureau. 2012.

[25] SoukAloun D Douangbouphaa V Phetsouvanh R Sibounheuang B Vongsouvat M Chanmala Ket al. Rotavirus diarrhea in hospitalized children under 5years of age in Vientiane, Lao PDR, 2009-2015. Vaccine. 2018;36:7878-82. 10.1016/j.vaccine.2018.04.004 29739715

[26] Bhutta ZA Black RE Brown KH Gardner JM Gore S Hidayat A Prevention of diarrhea and pneumonia by zinc supplementation in children in developing countries: pooled analysis of randomized controlled trials. Zinc Investigators’ Collaborative Group. J Pediatr. 1999;135:689-97. 10.1016/S0022-3476(99)70086-7 10586170

[27] Mulenga M Malunga P Bennett S Thuma P Shulman C Fielding K Folic acid treatment of Zambian children with moderate to severe malaria anemia. Am J Trop Med Hyg. 2006;74:986-90. 10.4269/ajtmh.2006.74.986 16760508

[28] Killilea DW Ames BN Magnesium deficiency accelerates cellular senescence in cultured human fibroblasts. Proc Natl Acad Sci U S A. 2008;105:5768-73. 10.1073/pnas.0712401105 18391207 PMC2311331

[29] Erhardt JG Estes JE Pfeiffer CM Biesalski HK Craft NE Combined measurement of ferritin, soluble transferrin receptor, retinol binding protein, and C-reactive protein by an inexpensive, sensitive, and simple sandwich enzyme-linked immunosorbent assay technique. J Nutr. 2004;134:3127-32. 10.1093/jn/134.11.3127 15514286

[30] Hess S, Barffour MA, Hinnouho GM. Lao Zinc Study Statistical Analyses Plan. Available: https://osf.io/5bq9c/. Accessed: 26 April 2019.

[31] Leroy JL Ruel M Habicht JP Frongillo EA Using height-for-age differences (HAD) instead of height-for-age z-scores (HAZ) for the meaningful measurement of population-level catch-up in linear growth in children less than 5 years of age. BMC Pediatr. 2015;15:145. 10.1186/s12887-015-0458-9 26444012 PMC4595313

[32] Barffour MA Hinnouho GM Kounnavong S Wessells KR Ratsavong K Bounheuang B Effects of daily zinc, daily multiple micronutrient powder, or therapeutic zinc supplementation for diarrhea prevention on physical growth, anemia, and micronutrient status in rural Laotian children: A randomized controlled trial. J Pediatr. 2019;207:80-89.e2. 10.1016/j.jpeds.2018.11.022 30580974 PMC6448681

[33] WHO. WHO. The treatment of diarrhoea: A manual for physicians and other senior health workers. 2005. Available: https://www.who.int/maternal_child_adolescent/documents/9241593180/en/. Accessed: 28 September 2016.

[34] Lazzerini M Ronfani L Oral zinc for treating diarrhoea in children. Cochrane Database Syst Rev. 2008;CD005436. 10.1002/14651858.CD005436.pub2 18646129

[35] Lazzerini M Oral zinc provision in acute diarrhea. Curr Opin Clin Nutr Metab Care. 2016;19:239-43. 26963581 10.1097/MCO.0000000000000276

[36] Fischer Walker CL Bhutta ZA Bhandari N Teka T Shahid F Taneja S Zinc supplementation for the treatment of diarrhea in infants in Pakistan, India and Ethiopia. J Pediatr Gastroenterol Nutr. 2006;43:357-63. 10.1097/01.mpg.0000232018.40907.00 16954960

[37] Brooks WA Santosham M Roy SK Faruque AS Wahed MA Nahar K Efficacy of zinc in young infants with acute watery diarrhea. Am J Clin Nutr. 2005;82:605-10. 10.1093/ajcn/82.3.605 16155274

[38] Dutta P Mitra U Dutta S Naik TN Rajendran K Chatterjee MK Zinc, vitamin A, and micronutrient supplementation in children with diarrhea: a randomized controlled clinical trial of combination therapy versus monotherapy. J Pediatr. 2011;159:633-7. 10.1016/j.jpeds.2011.03.028 21592508

[39] Faruque AS Mahalanabis D Haque SS Fuchs GJ Habte D Double-blind, randomized, controlled trial of zinc or vitamin A supplementation in young children with acute diarrhoea. Acta Paediatr. 1999;88:154-60. 10.1111/j.1651-2227.1999.tb01074.x 10102147

[40] Shankar AH Prasad AS Zinc and immune function: the biological basis of altered resistance to infection. Am J Clin Nutr. 1998;68:447S-63S. 10.1093/ajcn/68.2.447S 9701160

[41] Feikin DR Bigogo G Audi A Pals SL Aol G Mbakaya C Village-randomized clinical trial of home distribution of zinc for treatment of childhood diarrhea in rural Western kenya. PLoS One. 2014;9:e94436. 10.1371/journal.pone.0094436 24835009 PMC4023937

[42] Lassi ZS Haider BA Bhutta ZA Zinc supplementation for the prevention of pneumonia in children aged 2 months to 59 months. Cochrane Database Syst Rev. 2010;CD005978. 10.1002/14651858.CD005978.pub2 21154362

[43] Brooks WA Santosham M Naheed A Goswami D Wahed MA Diener-West M Effect of weekly zinc supplements on incidence of pneumonia and diarrhoea in children younger than 2 years in an urban, low-income population in Bangladesh: randomised controlled trial. Lancet. 2005;366:999-1004. 10.1016/S0140-6736(05)67109-7 16168782

[44] Tielsch JM Khatry SK Stoltzfus RJ Katz J LeClerq SC Adhikari R Effect of daily zinc supplementation on child mortality in southern Nepal: a community-based, cluster randomised, placebo-controlled trial. Lancet. 2007;370:1230-9. 10.1016/S0140-6736(07)61539-6 17920918 PMC2376970

[45] Wuehler SE Sempertegui F Brown KH Dose-response trial of prophylactic zinc supplements, with or without copper, in young Ecuadorian children at risk of zinc deficiency. Am J Clin Nutr. 2008;87:723-33. 10.1093/ajcn/87.3.723 18326612

[46] Ayede AI Kirolos A Fowobaje KR Williams LJ Bakare AA Oyewole OB A prospective validation study in South-West Nigeria on caregiver report of childhood pneumonia and antibiotic treatment using Demographic and Health Survey (DHS) and Multiple Indicator Cluster Survey (MICS) questions. J Glob Health. 2018;8:020806. 10.7189/jogh.08.020806 30254744 PMC6150611

[47] McDonald CM Manji KP Kisenge R Aboud S Spiegelman D Fawzi WW Daily zinc but not multivitamin supplementation reduces diarrhea and upper respiratory infections in Tanzanian infants: A randomized, double-blind, placebo-controlled clinical trial. J Nutr. 2015;145:2153-60. 10.3945/jn.115.212308 26203094 PMC4548161

[48] Mayo-Wilson E Imdad A Junior J Dean S Bhutta ZA Preventive zinc supplementation for children, and the effect of additional iron: a systematic review and meta-analysis. BMJ Open. 2014;4:e004647. 10.1136/bmjopen-2013-004647 24948745 PMC4067863

[49] Salam RA MacPhail C Das JK Bhutta ZA Effectiveness of Micronutrient Powders (MNP) in women and children. BMC Public Health. 2013;13 Suppl 3:S22. 10.1186/1471-2458-13-S3-S22 24564207 PMC3847468

[50] Paganini D Uyoga MA Kortman GAM Cercamondi CI Winkler HC Boekhorst J Iron-containing micronutrient powders modify the effect of oral antibiotics on the infant gut microbiome and increase post-antibiotic diarrhoea risk: a controlled study in Kenya. Gut. 2019;68:645-53. 10.1136/gutjnl-2018-317399 30448776

[51] Tang M Frank DN Hendricks AE Ir D Esamai F Liechty E Iron in micronutrient powder promotes an unfavorable gut microbiota in Kenyan infants. Nutrients. 2017;9:E776. 10.3390/nu9070776 28753958 PMC5537890

[52] Paganini D Zimmermann MB The effects of iron fortification and supplementation on the gut microbiome and diarrhea in infants and children: a review. Am J Clin Nutr. 2017;106:1688S-93S. 10.3945/ajcn.117.156067 29070552 PMC5701709

[53] Hess SY Wessells KR Hinnouho GM Barffour MA Sanchaisuriya K Arnold CD Iron status and inherited hemoglobin disorders modify the effects of micronutrient powders on linear growth and morbidity among young Lao children in a double-blind randomized trial. Br J Nutr. 2019;122:895-909. 10.1017/S0007114519001715 31303184 PMC7672373

[54] Breuer W Ghoti H Shattat A Goldfarb A Koren A Levin C Non-transferrin bound iron in Thalassemia: differential detection of redox active forms in children and older patients. Am J Hematol. 2012;87:55-61. 10.1002/ajh.22203 22125177

[55] Abbeddou S Hess SY Yakes Jimenez E Some JW Vosti SA Guissou RM Comparison of methods to assess adherence to small-quantity lipid-based nutrient supplements (SQ-LNS) and dispersible tablets among young Burkinabe children participating in a community-based intervention trial. Matern Child Nutr. 2015;11 Suppl 4:90-104. 10.1111/mcn.12162 25521188 PMC6860357

