# Supplementary material for: Effects of therapeutic zinc supplementation for diarrhea and two preventive zinc supplementation regimens on the incidence and duration of diarrhea and acute respiratory tract infections in rural Laotian children: A randomized controlled trial
Source: J Glob Health. 2020 Jun 27;10(1):010424. doi: 10.7189/jogh.10.010424 (PMC7321011; doi:10.7189/jogh.10.010424)
Supplement: Online Supplementary Document [file jogh-10-010424-s001.pdf]

Table S1: Modifying effects of child age on the responses to therapeutic zinc supplementation for diarrhea, daily preventive zinc supplementation, daily MNP, and placebo on diarrhea incidence, longitudinal prevalence, and average duration among young Laotian children.

| | TZ | PZ | MNP | Control | P-value |
|---|---|---|---|---|---|
| Treatment effects in children 6-17 months | 592 | 603 | 627 | 624 | |
| Diarrhea incidence (episodes per 100 days at risk) | 0.68 ± 003 | 0.64 ± 0.03 | 0.65 ± 0.03 | 0.63 ± 0.03 | 0.80 |
| Longitudinal prevalence (days with diarrhea per 100 days observed) | 1.87± 0.13 | 1.69 ± 0.12 | 1.59 ± 0.11 | 1.46± 0.10 | 0.08 |
| Diarrhea duration (days per episode) | 2.24 ± 0.07 | 2.27 ± 0.07 | 2.16 ± 0.07 | 2.11 ± 0.07 | 0.30 |
| | | | | | |
| Treatment effects in children 18-23 months | 259 | 259 | 225 | 228 | |
| Diarrhea incidence (episodes per 100 days at risk) | 0.40 ± 0.05 [a] | 0.47± 0.05[b] | 0.53 ± 0.05[b] | 0.58 ± 0.05[b] | 0.021 |
| Longitudinal prevalence (days with diarrhea per 100 days observed) | 0.74 ± 0.08[a] | 1.12 ± 0.13[b] | 0.99 ± 0.12[b] | 1.26 ± 0.14[b] | 0.005 |
| Diarrhea duration (days per episode) | 1.69± 0.10 | 1.97 ± 0.10 | 1.83 ± 0.10 | 1.99± 0.10 | 0.095 |

TZ=therapeutic zinc; PZ= preventive zinc; MN*P*=Micronutrient powder.

*All values adjusted for age, sex, district; values on the same row with different superscripts are significantly different (p<0.01).

Table S2: Age-stratified effects of the responses to therapeutic zinc supplementation for diarrhea, daily preventive zinc supplementation, daily MNP, and placebo on incidence of diarrhea episodes occurring after the first diarrhea treatment among rural Laotian children*.

| | **TZ** | **PZ** | **MNP** | **Control** | **Global P-value** |
|---|---|---|---|---|---|
| Children 6-17 month, N | 129 | 128 | 136 | 137 | |
| Acute diarrhea episodes per 100 days at risk | 0.27 ± 0.08 | 0.23 ± 0.07 | 0.27 ± 0.08 | 0.29 ± 0.08 | 0.93 |
| Children 18-23 months, N | 54 | 59 | 69 | 45 | |
| Acute diarrhea episodes per 100 days at risk | 0.07 ± 0.04 | 0.19 ± 0.10 | 0.17 ± 0.08 | 0.43 ± 0.22 | 0.07 |

All values adjusted for age, sex, district; Values on the same row with different superscripts are significantly different ($p<0.01$). Data prior to the first therapeutic supplementation excluded from the present analyses.
